# Coxiella burnetii Endocarditis: A Case Report

**DOI:** 10.7759/cureus.86147

**Published:** 2025-06-16

**Authors:** Carolina António Santos, Inês Parreira, Ana Alves Cardoso

**Affiliations:** 1 Department of Internal Medicine, Unidade Local de Saúde Santa Maria, Lisbon, PRT

**Keywords:** coxiella burnetii infection, endocarditis, mechanical valve, q-fever, zoonosis

## Abstract

Q fever is a zoonosis caused by the bacterium *Coxiella burnetii (C. burnetii)*, with goats, sheep, and cattle as the main reservoirs. Transmission to humans mainly occurs via inhalation of contaminated aerosols. Q fever can be acute or chronic, with infective endocarditis being the most common manifestation of the latter. It can be challenging to diagnose given the variety of clinical manifestations, and is usually diagnosed with the aid of serological tests. *C. burnetii* endocarditis is associated with a significant morbidity and mortality rate, and it is essential to make a rapid diagnosis of the condition in order to start targeted therapy as soon as possible. Treatment is based on antibiotic therapy, and surgical intervention may be necessary. We report a case of asymptomatic Q fever endocarditis and describe its diagnostic course, treatment, and follow-up.

## Introduction

Q fever is a zoonosis with a global incidence [1-3,4] that is often underdiagnosed; its clinical diagnosis can be difficult as it presents asymptomatically or with non-specific symptoms, as well as due to the difficulty in identifying the causative agent [2,4]. Q fever is caused by *Coxiella burnetii (C. burnetii)* [1-5], a Gram-negative bacterium [2,3]. The main reservoirs are goats, sheep [1,2,4], and cattle [1,4], although other hosts such as ticks [2-4], birds, and reptiles may also be involved [2,4]. Transmission to humans is mainly by inhalation of contaminated aerosols [1-4], via biological products from infected animals [2-4], or consumption of unpasteurised milk or milk products [2,4]. Human-to-human transmission is rare [2,4]. Clinically, Q fever presents in two forms: acute or chronic [1-5]. Diagnosis is often challenging due to the diversity of clinical manifestations [3,4] and the difficulty in isolating the causative agent [2-4]. This article focuses on the diagnostic approach to chronic endocarditis due to Q fever by presenting an illustrative clinical case.

## Case presentation

The patient was a 79-year-old female, partially dependent in activities of daily living, living in an urban area, with a history of cerebrovascular disease and valvular heart disease, who underwent valve replacement with an aortic mechanical prosthesis and a mitral bioprosthesis. She was allergic to penicillin and was on regular therapy with edoxaban and other drugs to control her comorbidities. She was admitted to the emergency department due to the sudden onset of dysarthria. She was seen by the neurology team, who noted a central facial nerve palsy and left hemiplegia consistent with a suspected stroke. The initial CT scan of the skull showed no acute intracranial lesions, but chronic microvascular leukoencephalopathy and various sequelae. Laboratory tests (Table 1) revealed hypochromic microcytic anaemia, thrombocytopenia, and elevated C-reactive protein (CRP). Based on the initial findings, the diagnosis of right middle cerebral artery ischaemic stroke was made, with no indication for immediate intervention.

**Table 1:** Laboratory findings

Variables	Admission (T0)	Discharge (T1)	Range
Hemoglobin (g/dL)	9.8	8.9	13.0-17.5
Mean corpuscular volume (fL)	72.8	71.9	80.0-97.0
Mean corpuscular hemoglobin (pg)	22.8	22.9	27-33
Leucocytes (x10^9^/L)	6.50	4.50	4.0-11.0
Platelets (x10^9^/L)	131	234	150-450
Aspartate aminotransferase (U/L)	35	28	0-40
Alanine aminotransferase (U/L)	15	18	0-41
Gamma-glutamyl transferase (U/L)	56	58	0-60
C-reactive protein (mg/dL)	8.97	1.11	<0.5
Total bilirrubin (mg/dL)	0.34	0.28	<1.2
Prothrombin time (seconds)	24.0	33.6	11.6
Activated partial thromboplastin time (seconds)	32.9	36.5	29
International normalized ratio	2.07	2.97	

During hospitalization, the neurological deficits resolved completely within 48-72 hours, and repeat imaging showed no new lesions. However, the etiological investigation of the stroke revealed echocardiographic (Video 1) findings suggestive of a valvular mass in the mechanical aortic prosthesis, raising the suspicion of a thrombus or infected vegetation.

**Video 1:** Transthoracic echocardiogram Transthoracic echocardiogram findings suggestive of a valvular mass in the mechanical aortic prosthesis, raising the suspicion of a thrombus or infected vegetation

Despite the absence of fever and significantly elevated inflammatory parameters (CRP ~2 mg/dL), the hypothesis of infective endocarditis could not be ruled out. A comprehensive serological work-up and serial blood cultures were performed without isolation of the pathogen. At the same time, an immunological and imaging study was performed to exclude autoimmune or paraneoplastic aetiologies of the endocarditis.

Initial investigations were negative for *Chlamydia psittaci, Legionella pneumoniae, Bartonella henselae, Mycoplasma pneumoniae, and Brucella *(using chemiluminescence techniques)and were negative for *Chlamydia pneumoniae* too (nucleic acid amplification tests techniques). Although serologies, using IFA (indirect immunofluorescence assay), were positive for *C. burnetti *with phase I IgG antibody titres above 1/800, confirming the diagnosis of Q fever endocarditis. The patient was therefore started on 100 mg of doxycycline twice daily with 200 mg of hydroxychloroquine three times daily and discharged with anticoagulation adjusted to vitamin K antagonists according to current recommendations. She is currently asymptomatic and is under regular serological monitoring.

## Discussion

Q fever, caused by *C. burnetii* [1-5], an obligate intracellular bacterium [1-4], presents clinical and diagnostic challenges [4]. While the acute form can be self-limiting [2] and often asymptomatic [2-4], the chronic form occurs in approximately 5% of acute infections [1-3] and can manifest years after initial exposure [3,4]. Major risk factors include valvular pathology, vascular prostheses or immunosuppression [2,4,5]. Our patient had one of these risk factors - valvular pathology. Chronic *C. burnetii *infection may be asymptomatic [1,3,4] or present with non-specific symptoms [1,3,4] such as fever [3,4], fatigue [4], weight loss or night sweats [3,4]. However, more severe forms may include endocarditis [2-4], vascular infections [3,4], arthritis [3,4], osteomyelitis [2-4], and hepatitis [4], among others [2,4]. Rare manifestations such as pulmonary embolism [4], myocarditis or haemophagocytic syndrome have also been described [2]. Endocarditis is the most common manifestation of chronic Q fever [1-5], followed by vascular prosthesis infections [1,4,5].

Our patient was clinically asymptomatic, with only the presence of a mechanical valve in the aortic position and a subfebrile temperature; hence, initially, we did not suspect an infectious disease. Although the transthoracic echocardiogram (which was performed for the etiologic investigation of the suspected stroke) findings were suggestive of vegetations, confirmation of endocarditis due to chronic Q fever is often difficult due to the non-specificity of the initial symptoms [2,3] and the difficulty in isolating the pathogen using conventional methods [2-4]. The diagnosis is based on a high index of clinical suspicion [4] and complementary laboratory methods [2-4], in particular IFA, which distinguishes between acute and chronic infection by phase I and phase II IgG antibody titres [1-4]. Higher phase I than phase II IgG titres indicate chronic infection [2-4], with values above 1/800 considered an important criterion for endocarditis [2-4]. Our patient's diagnosis was confirmed by high phase I IgG titres in IFA.

Treatment for chronic *C. burnetii* infection involves a combination of doxycycline 100 mg twice a day and hydroxychloroquine 200mg three times daily [2,4], and its duration varies based on the organ involved [4]. Our patient had only a cardiac valve involved. For prosthetic valve endocarditis, therapy is recommended for at least 24 months [4], as in the case reported here. Regular monitoring is essential, both because of the need to adjust therapy and to detect potential adverse effects, such as the retinal toxicity associated with hydroxychloroquine, which requires regular ophthalmological assessment every six months [4]. Patient monitoring is based on clinical and laboratory (antibody titres) every month [4].

*C. burnetii* endocarditis has a high morbidity and mortality rate [3,4], particularly due to the possibility of heart failure [1,2]. In addition, the risk of recurrence after cessation of therapy is significant, particularly in patients with mechanical valves [3]. Therefore, regular clinical [4] and serological follow-up is essential [2,4] to assess the efficacy of treatment [4] and the ideal time to stop treatment [2]. In cases of advanced chronic infection, with severe heart failure or valvular abscess, antibiotic therapy is not sufficient, and surgery is the best therapeutic option [2].

In the case described, the patient will remain on combination therapy with regular serological monitoring. Progress is assessed on the basis of a sustained reduction in phase I IgG antibody titres, with the expectation that therapy will be discontinued when values below 1:2 or a fourfold reduction from baseline titres are achieved [2,4]. The patient currently remains asymptomatic, and the phase I IgG antibody titres are already lower.

## Conclusions

We discussed a case of chronic Q fever in an asymptomatic patient without epidemiology, although the patient had risk factors for it. Q fever is a zoonosis caused by *C. burnetii*, which is transmitted to humans mainly by inhalation of contaminated aerosols, and it can be acute or chronic, with endocarditis being the most common manifestation of the chronic form. Diagnosis of chronic infection is difficult and is based on clinical suspicion and serological methods. Our patient was an elderly female with a prosthetic cardiac valve who was admitted to the hospital with a suspected stroke. During the etiological investigation of stroke, a transthoracic echocardiogram was performed with aspects suggestive of prosthetic valve endocarditis. The patient remained asymptomatic during the hospitalization, with no physical alteration at observation and only a discrete elevation of inflammatory parameters and subfebrile temperature. In order to identify the etiology of the endocarditis, different laboratory tests were performed, with a positive result in IgG antibody titres in IFA for *C.* *burnetti, *prompting a diagnosis of endocarditis due to *C.* *burnetti *in an asymptomatic patient with a prosthetic valve. The patient was started on adequate antibiotherapy and is under clinical and laboratory monitoring every month. The patient remains asymptomatic without any complications of the endocarditis or of the treatment.

## References

[1] Heydari AA, Mostafavi E, Heidari M, Latifian M, Esmaeili S (2021). Q fever endocarditis in northeast Iran. Case Rep Infect Dis.

[2] Ullah Q, Jamil T, Saqib M, Iqbal M, Neubauer H (2022). Q fever-a neglected zoonosis. Microorganisms.

[3] Grey V, Simos P, Runnegar N, Eisemann J (2023). Case report: a missed case of chronic Q fever infective endocarditis demonstrating the ongoing diagnostic challenges. Access Microbiol.

[4] (2025). CDC: Diagnosis and management of Q fever — United States. http://www.cdc.gov/mmwr/preview/mmwrhtml/rr6203a1.htm.

[5] de Lange MM, Scheepmaker A, van der Hoek W, Leclercq M, Schneeberger PM (2019). Risk of chronic Q fever in patients with cardiac valvulopathy, seven years after a large epidemic in the Netherlands. PLoS One.

